# IL-17 induces reactive astrocytes and up-regulation of vascular endothelial growth factor (VEGF) through JAK/STAT signaling

**DOI:** 10.1038/srep41779

**Published:** 2017-03-10

**Authors:** Tao You, Yihui Bi, Jun li, Mingkai Zhang, Xuezhou Chen, Keke Zhang, Jun Li

**Affiliations:** 1 College of Pharmacy, Anhui Medical University, Hefei, China; 2 Department of Orthopaedics, The First Affiliated Hospital, Anhui Medical University, Hefei, China; 3 Department of Orthopaedics, The Second Affiliated Hospital, Anhui Medical University, Hefei, China

## Abstract

Spinal cord injury is a grave neurological disability resulting in neuron degeneration and permanent paralysis. The inflammation triggered by the injury would promote the spinal cord lesion in turn. Activated astrocytes during inflammatory response could promote glial scar formation and contribute to the progression of the spinal cord injury. Interleukin 17 (IL-17) was upregulated in inflammatory responses to contusion or compression of the spinal cord. in this study, IL-17 could induce reactive astrocytes which was indicated by a well-known hallmark glial fibrillary acidic protein (GFAP) *in vitro* and *in vivo*. Moreover, we demonstrated that the upregulation of VEGF was induced by IL-17 human astrocytoma cells. In our further investigation, IL-17 induced the expression of VEGF in spinal cord injury by activating JAK/STAT signaling pathway both *in vitro* and *in vivo*. In addition, we also found that IL-17 significantly changed tissue preservation and residual urine volumes and blood-spinal cord-barrier integrity *in vivo*. This newly found IL-17-JAK/STAT-VEGF axis improves our understanding of the molecular mechanism of spinal cord injury during inflammatory response and provides another potential target of spinal cord injury.

The spinal cord is a key component of the central nervous system, connecting the peripheral nervous system and the brain. The spinal cord injury (SCI) is a damage to normal function of spinal cord, induced by the primary injury and the secondary injury^1^. The primary injury arises from the traction and compression forces. The secondary injury is initiated by cerebral trauma and ischaemia following the primary injury. The spinal cord injury is mainly derived from the mechanical damage to the spinal cord which is initiated by the primary injury^2^. Injury to the spinal cord elicits a range of patho-physiological processes, including metabolic disturbance of the extracellular matrix (ECM), inflammatory response, as well as lesion-activated proliferation of microglia and astrocytes^3^. Astrocytes play a decisive role in spinal cord injury, exerting well-known detrimental effects that lead to a glial scar formation^4^. As previously demonstrated, vascular endothelial growth factor (VEGF) was significantly upregulated in spinal cord injured tissue, and was associated with astrocytic and microglia activation around the site of spinal cord injury^5^. However, the role of upregulated VEGF in spinal cord injury remains unclear. Further studies are needed to address whether upregulation of VEGF would induce spinal cord injury.

In spinal cord injury, many factors are involved in the upregulation of VEGF and the activation of microglia and astrocytes. In *in vitro* model cells system, an elevation of VEGF was observed in gliocytes after Prostaglandin F2alpha (PEF2α) injection^6^. It was demonstrated that hypoxia could induce the expression of VEGF in spinal cord injury^7^. Further studies on the molecular mechanism between hypoxia and VEGF showed that HIF-2α preferentially regulated the expression and promoter activity of cited2, which would lead to the expression of VEGF^8^. IL-17 plays an important role in inflammation, as it is an important pro-inflammatory factor produced by Th17^9^. Moreover, IL-17 is upregulated in the spinal cord injury, and plays a central role in spinal cord neuro-inflammation^10^. IL-17 has been shown to synergize with IL-1β, IL-22, IFN-γ, TNF-α and other cytokines *in vivo*^11^. Notably, IL-17 is vigorously involved in mediating pro-inflammatory responses via the induction of many other cytokines, including IL-6, granulocyte-macrophage colony-stimulating factor (GM-CSF), IL-1β, TGF-β, TNF-α and chemokines, including IL-8 and monocyte chemotactic protein-1 (MCP-1), from many cells^12^. Osteoarthritis (OA) is a multifactorial disease which is characterized by an evident inflammation: synovial hypertrophy, proliferation of synovial lining cells. IL-17 in OA cells causes the increasing expression of VEGF^13^. The role of IL-17 and VEGF that played in spinal cord injury, as well as the detailed mechanism of VEGF in spinal cord injury, would be revealed by further investigation.

The regulatory mechanism varies across different cells or different models. Angiogenesis plays a critical role in the invasion, metastasis and progression of hepatocellular carcinoma. In MHCC97H cell which is recognized as a typical HCC cell line with high metastasis capacity, Janus kinase/signal transducer and activator of transcription (JAK/STAT) signaling pathway would influence the expression of VEGF^14^. VEGF could be induced by many receptor and intracellular oncogenic proteins like JAK/STAT that commonly activated in cancer^15^. In vascular smooth muscle cells, STAT1 and STAT3 plays opposing roles in regulating the expression of VEGF. STAT1 inhibits VEGF expression, while STAT3 could promote the expression of VEGF^16^. Moreover, in human retinal pigment epithelial cell, JAK/STAT signaling pathway influences VEGF as well, since the STAT1 pathway inhibitor downregulated the secretion of VEGF^17^.

The mechanism that IL-17 modulates VEGF in spinal cord injury remains unclear. However, JAK/STAT signaling pathway could be activated by the pro-inflammatory cytokine IL-17. The first evidence linking IL-17 and JAK/STAT signaling pathway was presented by Subramanlam that IL-17 induced JAK/STAT signaling pathway in a time-dependent manner^18^. In fibroblast-like synoviocytes, IL-17 could promote the JAK/STAT signaling pathway, and the IL-17/STAT pathway plays a critical role in rheumatoid arthritis^19^. In this study, we illustrate that IL-17 could modulate the expression of VEGF by activating the JAK/STAT signaling pathway *in vitro and in vivo*, which will further promote the activation of astrocytes and provide a better understanding of spinal cord injury.

## Results

### IL-17 induces reactive astrocytes

**Figure 1:**
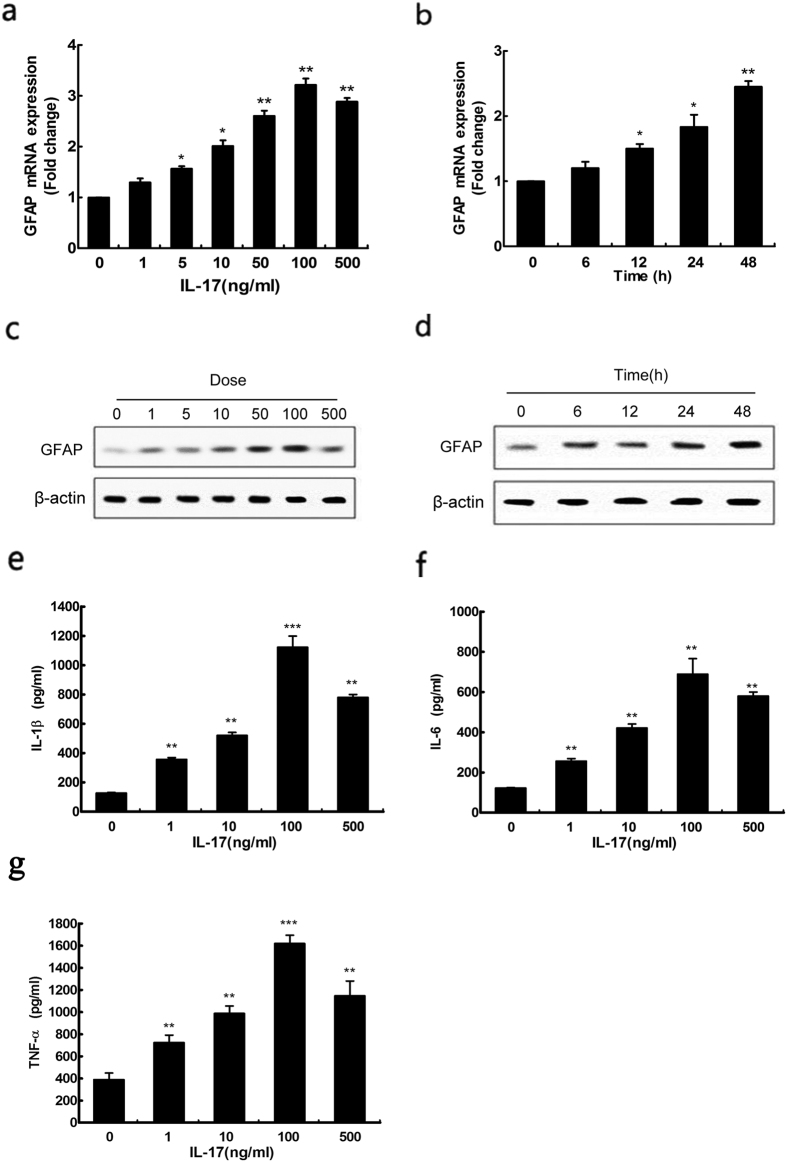
Evidence that IL-17 induces reactive astrocytes relevant protein GFAP in human astrocytoma cell lines. Quantitative reverse transcription-PCR analysis of GFAP genes. U251 cells treated with various concentration of IL-17 for 24 h (**a**) and 100 ng/ml IL-17 for different hours (**b**). The expressions of GFAP protein were determined by western blot. Cells were treated with various concentrations of IL-17 for 24 h (**c**) or incubated with 100 ng/ml IL-17 for different hours (**d**). The expressions of proinflammatory cytokines IL-1β (**e**), IL-6 (**f**), TNF-α (**g**) were determined by ELISA. Cells were treated with various concentrations of IL-17 for 24 h. Results are expressed as the mean ± S.D. from three independent experiments. *P < 0.05 versus control, **P < 0.01 versus control, ***P < 0.001 versus control.

Overexpression of glial fibrillary acidic protein (GFAP) by reactive astrocytes is perhaps the best known hallmark of reactive astrocytes^20^. Therefore, we first investigated the effect of IL-17 on GFAP in human astrocytoma cell lines, by incubating U251 cells with various concentrations of IL-17 for 24 h (Fig. 1a and c) and 100 ng/ml IL-17 for different hours (Fig. 1b and d). Q-PCR and western blot demonstrated that the expression of GFAP was upregulated at mRNA and protein levels with increasingly concentrations of IL-17, and increased in a time-dependent manner during the 48-hour treatment period. Furthermore, human astrocytoma cells were challenged with 0, 1, 10, 100, or 500 ng/ml of IL-17 for 24 h, and then the production of pro-inflammatory cytokines IL-1β, IL-6 and TNF-α (Fig. 1e,f and g) were determined by ELISA. We also demonstrated that the elevated IL-1β, IL-6 and TNF-α induction by IL-17 in U251 cells. These data suggested that IL-17 may induces reactive astrocytes.

### IL-17 stimulates VEGF production by human astrocytoma cell

**Figure 2:**
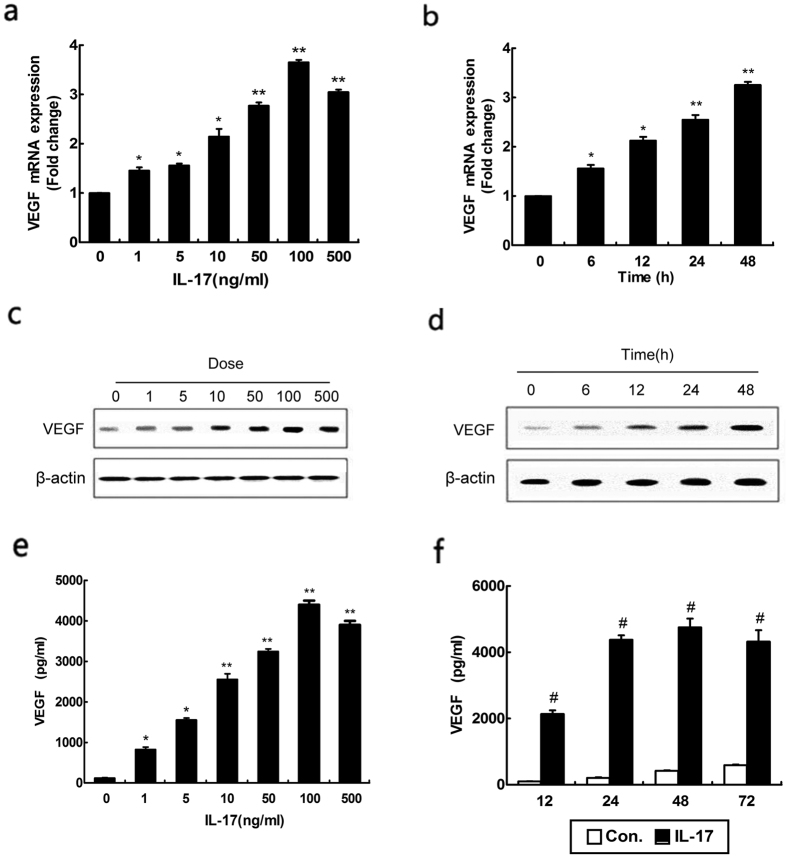
Effect of IL-17 on VEGF production in human astrocytoma cell lines. Quantitative reverse transcription-PCR analysis of VEGF genes. U251 cells treated with various concentration of IL-17 for 24 h (**a**) and 100 ng/ml IL-17 for different hours (**b**). Results are expressed as the mean ± S.D. from three independent experiments. *P < 0.05 versus control, **P < 0.01 versus control. The statistical differences were calculated by by the Student’s t-test. The expressions of VEGF protein were determined by western blot. Cells were treated with various concentrations of IL-17 for 24 h (**c**) or incubated with 100 ng/ml IL-17 for different hours (**d**). Results are expressed as VEGF secreted by cells incubated for 72 h with different concentrations of IL-17 (**e**) or treated with PBS as control and 100 ng/ml IL-17 for different hours (**f**). Results are expressed as the mean ± S.D. from three independent experiments. *P < 0.05 versus control, **P < 0.01 versus control, ^#^P < 0.01 versus PBS treatment.

It has been reported that IL-17 can enhance VEGF secretion by dedifferentiated OA chondrocytes^13^. However, the effect of IL-17 on VEGF in SCI remains elusive. To investigate whether IL-17 influences VEGF production in human astrocytoma cells, U251 cells were also incubated with various concentrations of IL-17 for 24 h (Fig. 2a and c) and 100 ng/ml IL-17 for different hours (Fig. 2b and d). Q-PCR and western blot demonstrated that the expression of VEGF was elevated at mRNA and protein levels with increasingly concentrations of IL-17, and increased in a time-dependent manner during the 48-hour treatment period. Consistently, VEGF secretion was observed to be significantly raised in U251 cells when detected using enzyme-linked immunosorbent assay (ELISA) with increasingly concentrations of IL-17 and was higher than control during all the period of treatment. Taken together, these results showed that the duration of IL-17 stimulation may contribute to the production of VEGF by reactive astrocytes.

### IL-17-induced activation of JAK/STAT signaling pathway to VEGF production

**Figure 3:**
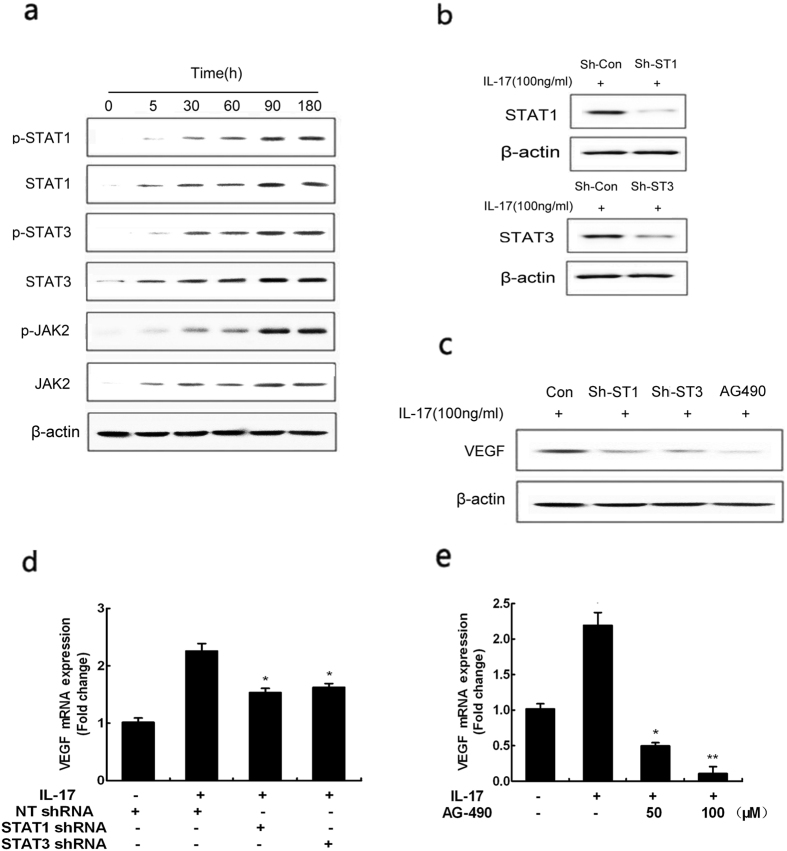
Effect of IL-17 on VEGF production correlated with JAK/STAT signaling pathway in human astrocytoma cell lines. The expressions of JAK/STAT signaling pathway protein p-STAT1, STAT1, p-STAT3, STAT3, p-JAK2, JAK2 were determined by western blot. Cells were treated with 100 ng/ml concentrations of IL-17 for different hours (**a**). Efficiency of ShRNA for STAT1 and STAT3 was tested by immunoblotting using lysates from cells treated with NT shRNA or STAT1 shRNA or STAT3 shRNA and 100 ng/ml concentrations of IL-17 for 48 h (**b**) or incubated with 100 ng/ml concentrations of IL-17 and NT shRNA, STAT1 shRNA, STAT3 shRNA, JAK2 inhibitor AG490 for 48 h (**c**) Quantitative reverse transcription-PCR analysis of VEGF genes. Cells were treated with 100 ng/ml concentration of IL-17 or non-stimulated conditions for 48 h. Meanwhile cells were incubated with NT shRNA or STAT1 shRNA or STAT3 shRNA (**d**). Cells were treated with 100 ng/ml concentration of IL-17 or non-stimulated conditions and JAK2 inhibitor AG490 for 48 h (**e**). Results are expressed as the mean ± S.D. from three independent experiments. *P < 0.05 versus NT shRNA, **P < 0.01 versus NT shRNA.

To determine whether IL-17-dependent mechanism contributes to activation of VEGF production, we investigated activation of JAK/STAT signaling following 100 ng/ml IL-17 stimulation by western blot analysis in human astrocytoma cells. Our results showed that IL-17 promoted the expression of phospho-STAT1/STAT1, phospho-STAT3/STAT3 and phospho-JAK2/JAK2 in a time-dependent manner during the 180-hour treatment period (Fig. 3a). To further determine whether the activation of JAK/STAT signaling pathway involved in VEGF expression, we found that VEGF effectively decreased at protein and mRNA levels by utilizing shRNAs against STAT1 or STAT3 although in the presence of IL-17, compared with a control shRNA (Fig. 3b,c and d). Moreover, cells were treated simultaneously with a JAK2 inhibitor AG490 and 100 ng/ml IL-17 for 48 h, and western blotting analysis and q-PCR revealed that VEGF expression was suppressed dramatically at protein and mRNA levels. Collectively, these data demonstrated the contribution of IL-17-induced activation of JAK/STAT signaling pathway to VEGF production.

### IL-17 modulates VEGF expression in a JAK/STAT-dependent manner

**Figure 4:**
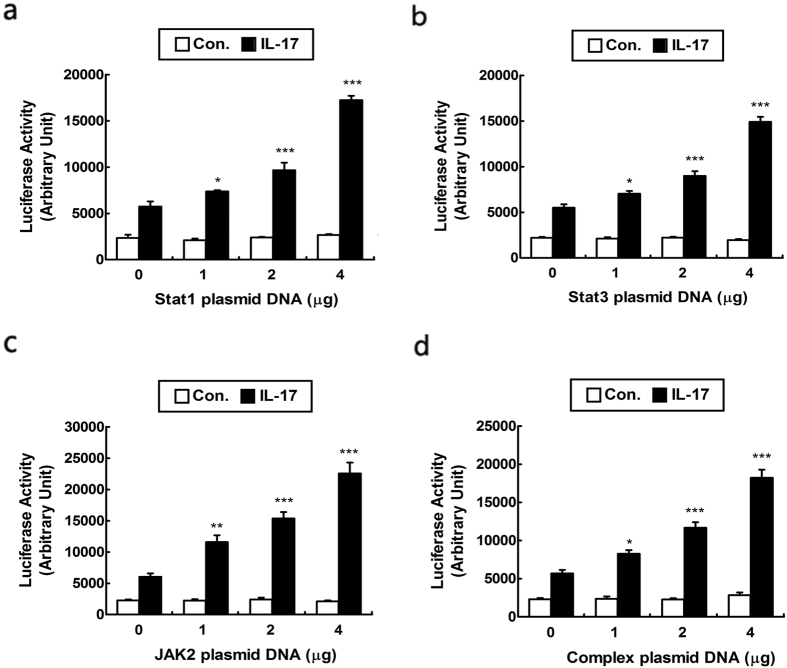
IL-17 enhanced the transcriptional activity of VEGF promoter by regulation of STAT1, STAT3 and JAK2. Cells were incubated with or without IL-17 for 48 h after transfection with various concentrations of STAT1 (**a**), STAT3 (**b**), JAK2 (**c**) or complex (**d**) plasmid DNA and 1 μg of VEGF promoter luciferase reporter plasmid, incubated with PBS as negative control. After incubating with or without IL-17, cell were prepared for luciferase assays, which were measured by using a luciferase reporter assay system. Values were normalized as the arbitrary luciferase activity. Results are expressed as the mean ± S.D. from three independent experiments. *P < 0.05 versus control, **P < 0.01, ***P < 0.001 versus shikonin independently treatment.

To further elucidate the role of JAK/STAT signaling pathway in the regulation of IL-17-mediated VEGF promoter activity, we performed co-transfection with various concentrations of STAT1, STAT3, JAK2 or complex plasmid DNA and 1 μg of VEGF promoter luciferase reporter plasmid after cells were incubated with or without IL-17 for 48 h (Fig. 4a–c). The results demonstrated that STAT1, STAT3 or JAK2 considerably increased luciferase expression in a dose-dependent manner directed by VEGF promoter after IL-17 stimulation. It suggested that IL-17 enhanced the transcriptional activity of VEGF promoter in a JAK/STAT-dependent manner.

### VEGF exert an important role in IL-17-induced reactive astrocytes

**Figure 5:**
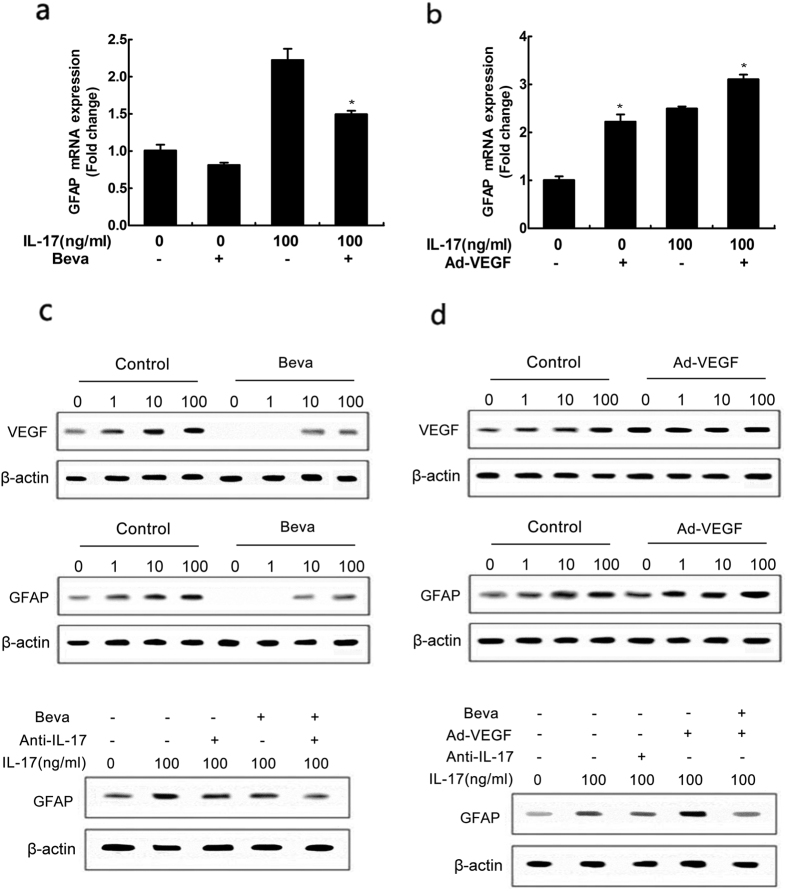
Effect of VEGF on reactive astrocytes. Quantitative reverse transcription-PCR analysis of GFAP genes. Cells were treated with 100 ng/ml concentration of IL-17 or non-stimulated conditions for 48 h after incubating with or without 100 ug/ml concentration of VEGF inhibitor bevacizumabor (**a**) or infecting with or without adenovirus vector containing VEGF (**b**). Results are expressed as the mean ± S.D. from three independent experiments. *P < 0.05 versus control group, ^#^P < 0.05 versus 100 ng/ml concentration of IL-17 group. The statistical differences were calculated by the Student’s t-test. The expressions of VEGF and GFAP were determined by western blot. Immunoblotting for VEGF and GFAP using lysates from cells were treated with various concentrations of IL-17 for 48 h after incubating with or without 100 ug/ml concentration of VEGF inhibitor bevacizumabor and (**c**) or infecting with or without adenovirus vector containing VEGF (**d**) and incubated with IL-17 inhibitor or VEGF inhibitor bevacizumabor at the same time.

**Figure 6:**
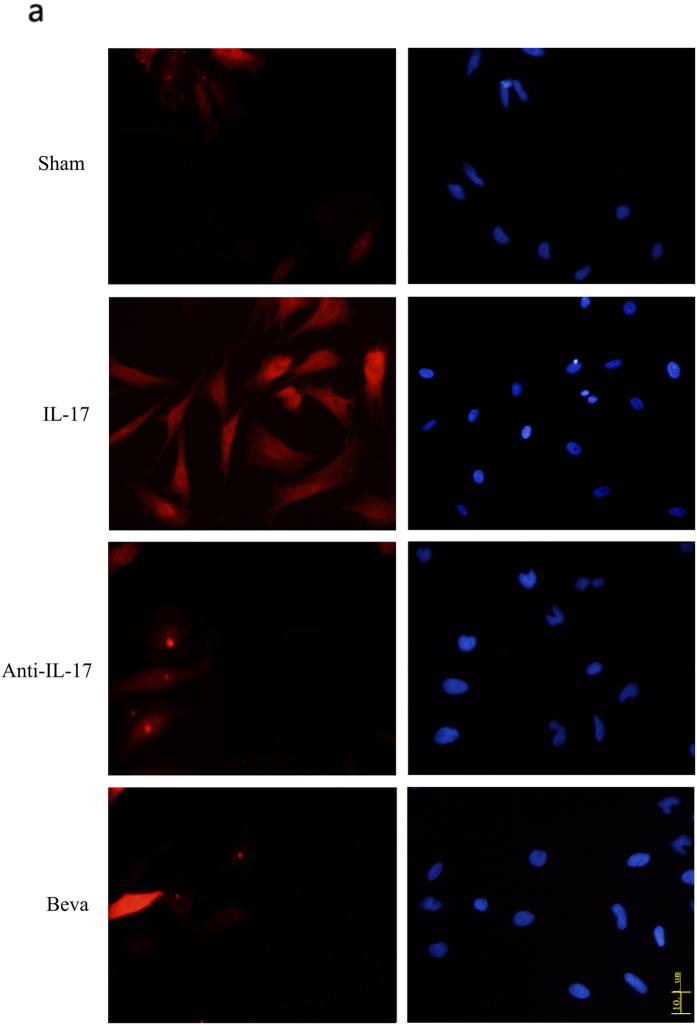
The effect of anti-IL-17 and Beva on GFAP expression in reactive astrocytes. Cells were treated with 100 ng/ml concentration of IL-17 or non-stimulated conditions or anti-IL-17 and Beva for 24 h.

As illustrated above, IL-17 induces reactive astrocytes to promote VEGF expression by activating JAK/STAT signaling pathway. However, the exact role whereby VEGF drives reactive astrocytes remains elusive. For this purpose, we utilized VEGF inhibitor bevacizumabor and adenovirus vector containing VEGF to suppress and increase VEGF expression, respectively (Fig. 5c and d, upper row). Our results showed that bevacizumabor exerted a significant suppressive effect on IL-17-induced GFAP expression as well as VEGF (Fig. 5a and c), while adenovirus vector containing VEGF exhibited effectively GFAP upregulation after IL-17 stimulation (Fig. 5b and d). The involvement of VEGF in IL-17-induced reactive astrocytes and GFAP production was confirmed by treating cells with a IL-17 inhibitor or bevacizumabor simultaneously. Western blot analysis showed that IL-17-promoted GFAP expression attenuated by IL-17 inhibitor and bevacizumabor (Fig. 5c, lower row). Furthermore, the re-introduction VEGF reversed inhibitory effect of bevacizumabor on GFAP expression (Fig. 5d, lower row). Consisting with the western blot results, immunofluorescence staining demonstrated a considerable increase of GFAP protein expression stimulated by IL-17, and GFAP expression attenuated by IL-17 inhibitor or bevacizumabor (Fig. 6a and Supplementary Figure 1).

### IL-17 induced the expression of VEGF in spinal cord injury by activating JAK/STAT signaling pathway *in vivo*

**Figure 7:**
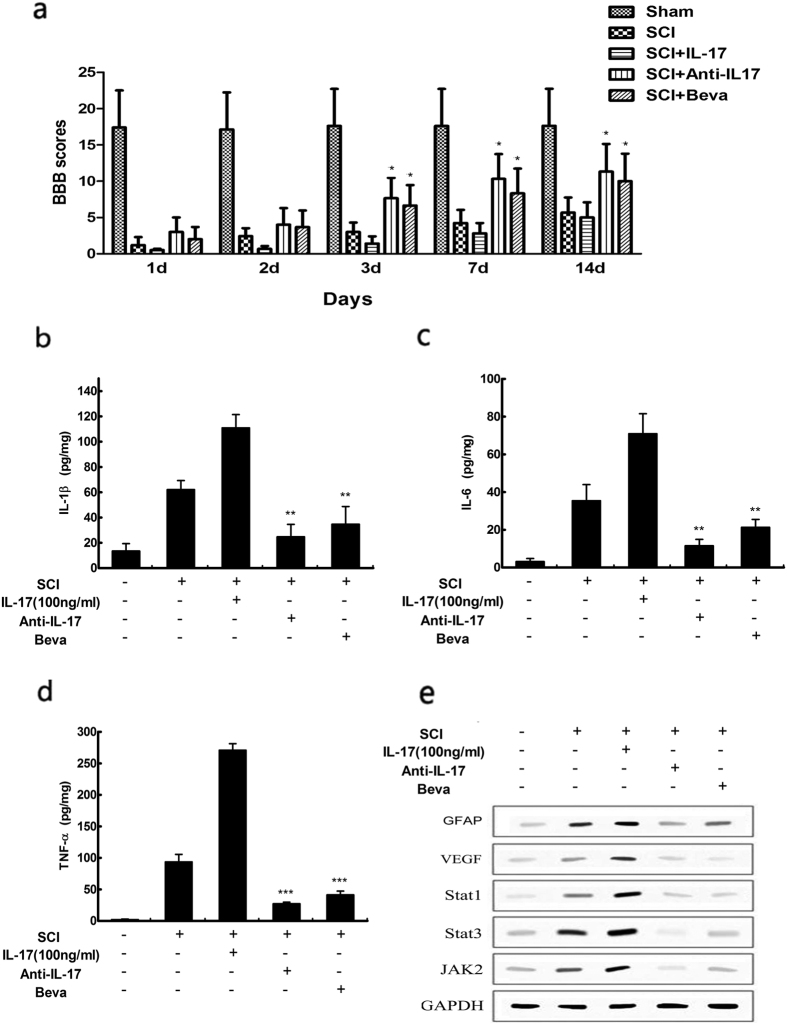
Basso, Beattie and Bresnahan (BBB) scores of rats in each group at different time points after injury. The BBB scores for hindlimb function were evaluated at 1, 2, 3, 7, and 14 days after sham surgery or spinal cord injury (**a**). n = 6/group for all the groups. Quantification data of IL-1β (**b**), IL-6 (**c**), TNF-α (**d**) production in the spinal cord at 72 h after injury, as assessed by ELISA. The expressions of JAK/STAT signaling pathway protein STAT1, STAT3, JAK2 were determined by western blot. spinal cord were prepared after treatment with IL-17 or anti-IL-17 and Beva for 72 hours (**e**). Results are expressed as the mean ± S.D. from three independent experiments. *P < 0.05 versus control, **P < 0.01 versus SCI group, ***P < 0.001 versus SCI group.

We tested whether IL-17 treatment would affect locomotor recovery after SCI by comparing controls and animals treated with IL-17 inhibitor or bevacizumabor at the time of injury. Rats were assessed by the BBB locomotor scale for 14 days post-injury and treatment. Testing of locomotor function showed that BBB scores of IL-17-treated animals were significantly lower than SCI controls or animals treated with IL-17 inhibitor and bevacizumabor (Fig. 7a). The local inflammation during locomotor recovery of SCI was evaluated by expression of IL-1β, IL-6 and TNF-α at 72 h post-injury. Expression of IL-1β, IL-6 and TNF-α protein was measured using ELISA. Similarly, IL-17 dramatically elevated IL-1β, IL-6 and TNF-α expression compared with the injury control groups and treated with IL-17 inhibitor or bevacizumabor (Fig. 7b–d). Moreover, we further analysed the expression of JAK/STAT signaling pathway protein STAT1, STAT3, JAK2 in the spinal cord by western blot. As expected, IL-17 promoted GFAP and VEGF expression as well as activating JAK/STAT signaling pathway compared with the injury control groups and treated with IL-17 inhibitor or bevacizumab (Fig. 7e). In summary, these data demonstrated that VEGF exert an important role in IL-17-induced reactive astrocytes.

### IL-17 inhibited the recovery of the animals tissue repair and residual urine volumes

**Figure 8:**
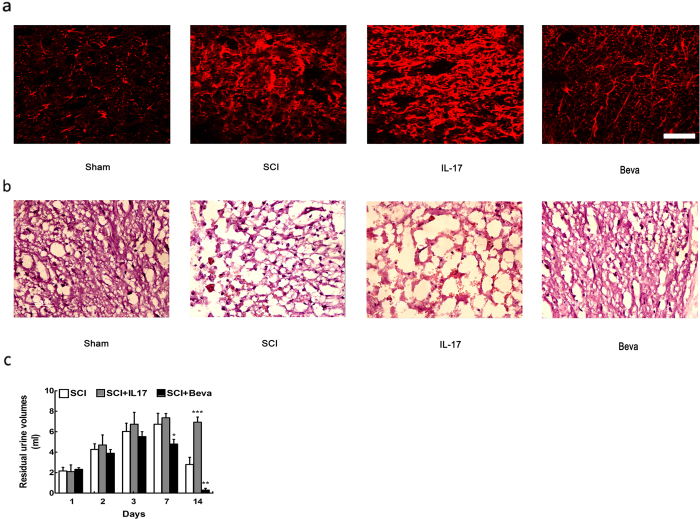
IL-17 damaged tissue preservation. (**a**) GFAP immunoreactivity to the injury site at 7 days post-injury. (**b**) H&E staining was used to visualize tissue in spinal segments. (**c**) Residual urine was collected and the volumes recorded for 14 days post-injury. Data presented as mean ± SD. *P < 0.05 versus SCI group, **P < 0.01 versus SCI group, ***P < 0.001 versus SCI group. Scale bars: 100 um.

The HE staining focused on the damaged tissue at 3 day postinjury were collected to determine the effect of IL-17 on the spinal cord recovery (Fig. 8b). The results showed manifestations of injury were obviously reduced after treatment with VEGF inhibitor, compared with the SCI group. There was haemorrhage, loose structure and neutrophil infiltration appeared in the IL-17 treatment. However, the injury was remarkably reduced after VEGF inhibitor treatment compared with SCI treated group. In addition, we also assessed residual urine volumes in various SCI groups. We found an significant decrese in residual urine valumes in the SCI plus bevacizumab group after 7 days of SCI, compared with IL-17 treated animals (Fig. 8c). However, residual urine volumes in the group of IL17 were remarkable increase at 14 day compared to SCI group. Decrease in residual urine valumes at group of SCI plus bevacizumab may reveals that animal recovery in residual urine volumes after treatment with VEGF inhibitor.

### Il-17 enhanced astrocyte reactivity *in vivo*

**Figure 9:**
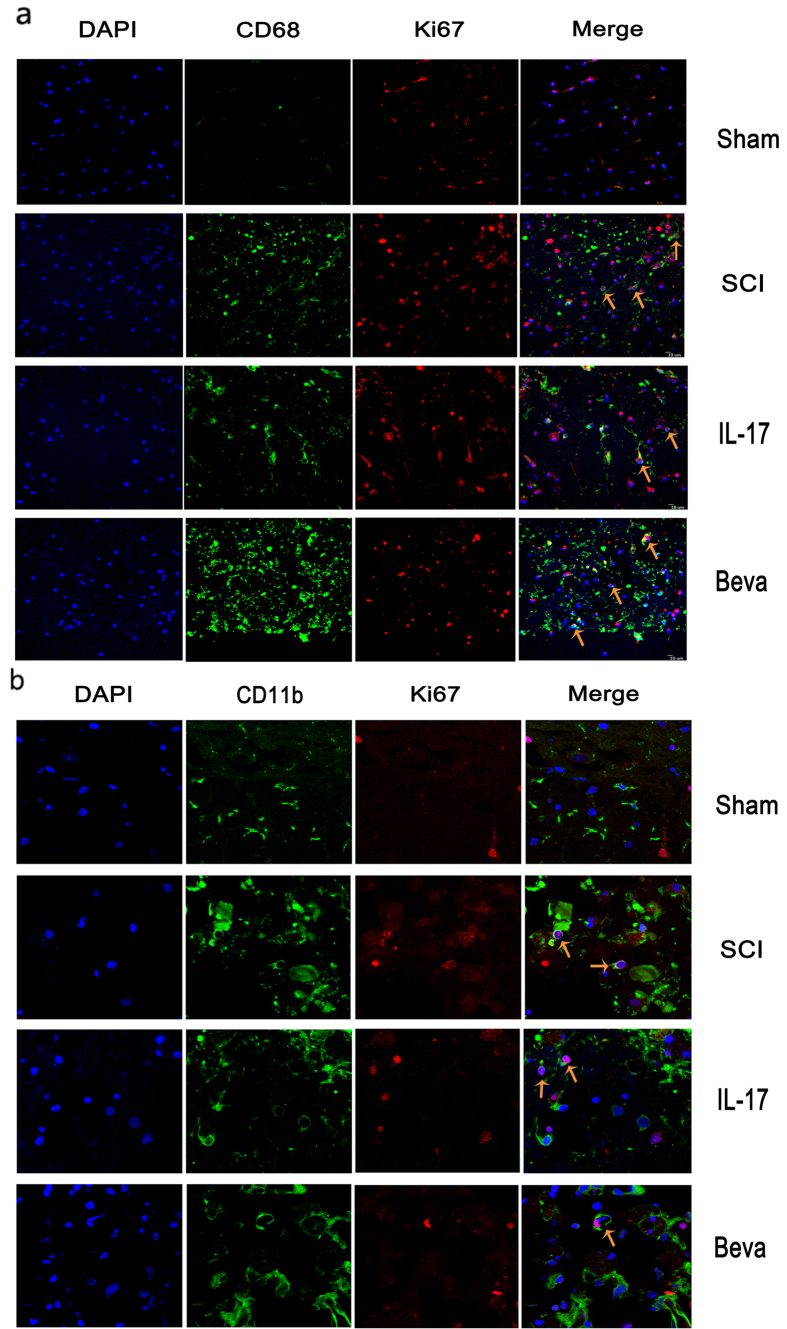
IL-17 have no significant effect on the proliferation of micrographs and microglia. (**a**) Co-labeled CD68 immunoreactive and Ki67 marker at day 7 post-injury. (**b**) Co-labeled CD11b immunoreactive and Ki67 marker at day 7 post-injury. Arrows represent the proliferation of positive cells.

IL-17 enhanced GFAP immunoreactivity compared with SCI group, while GFAP intensity decreased in the VEGF inhibitor treated group (Fig. 8a). CD68 and CD11b immunoreactivity were used to assess activated macrophages and microglia. We found that IL-17 treatment have not changed the CD68 immunoreactive activated macrophages compared to SCI treatment group, as well as VEGF inhibitor treatment. Furthermore, we co-labeled CD68 positive cells with the proliferation marker Ki67 (Fig. 9a). The results showed that IL-17 had no significant impact on proliferation of CD68 positive cells at day 7 post-injury (Fig. 9a). Moreover, we investigated whether IL-17 affected the proliferation of CD11b positive cells, and found that there was no significant effect (Fig. 9b).

### IL-17 decreased Blood-spinal Cord-barrier Integrity

**Figure 10:**
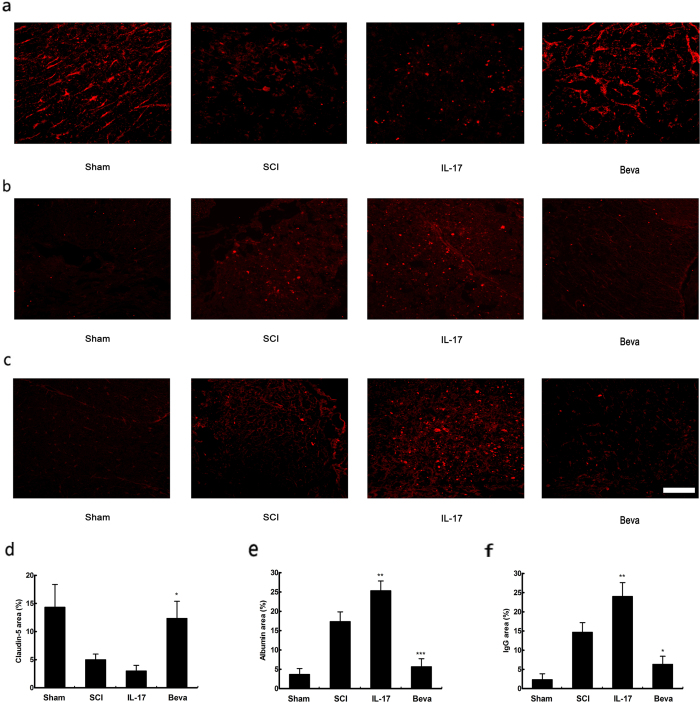
IL-17 damaged BSCB integrity. (**a**,**d**) Claudin-5 immunoreactivity was detected and compared at 7 days post-injury. (**b**,**e**) Albumin immunoreactivity was detected and compared at 7 days post-injury. (**c**,**f**) IgG immunoreactivity was detected and compared at 7 days post-injury. Data presented as mean ± SD. *P < 0.05 versus SCI group, **P < 0.01 versus SCI group, ***P < 0.001 versus SCI group. Scale bar: 100 um.

To determine the effects of IL-17 on blood spinal cord barrier integrity, we evaluated the expression of the junction protein claudin-5 at day 7 after injury. The results revealed more intense claudin-5 immunoreactivity within the VEGF inhibitor-treated rats compared with SCI-treated rats, whereas IL-17 treated group significant decrease with the expression of claudin-5 compared with SCI-treated rats (Fig. 10a and d). In addition, to assess BSCB integrity, we also quantified leakage of albumin and IgG proteins. We found VEGF inhibitor remarkably reduced leakage of the two proteins compared with SCI-treated group (Fig. 10e and f). However IL-17 obviously enhanced albumin and IgG leakage into spinal cord segments compared with SCI-treated rats (Fig. 10e and f).

## Discussion

Spinal cord injury is a grave neurological disability that would have a significant impact on the individual, the family and the society as well^21^. Spinal cord injury generally leads to neuron degeneration under the injury and results in permanent paralysis. Numerous neuroglia cells which play a decisive role in many aspects of neurons definitely participate in the process of spinal cord injury, such as astrocytes that contribute to glial scar formation^22^,^23^.

Contusion or compression of spinal cord cause acute central hemorrhagic necrosis accompanied by glial activation and inflammatory infiltration^24^. The distribution of microglia, astrocytes and T-lymphocytes was well characterized in the spinal cord injury resulting from the primary injury^25^. Like many other tissue damages, spinal cord injury triggers inflammatory cascade, which may in turn promotes the spinal cord lesions in a post-traumatic manner^26^. The inflammation induced by spinal cord injury may result in further degeneration in recovery, due to the scar tissue and necrosis or apoptosis. The inflammatory response induced after injury extends the region of necrosis and apoptosis beyond the actual impact site, damaging a much larger area and aggravating the loss of function^27^. IL-17 is one of the most famous pro-inflammatory factors produced by Th17 cells and plays a crucial role in inflammation. IL-17 is widely involved in all aspects of either innate or adaptive immunity and would promote autoimmune pathology^28^. IL-17 regulates inflammatory response through synergistic signaling, with other cytokines, including TNFα, IL-1β^29^,^30^. Given that IL-17 is an important pro-inflammatory factor, it is not surprising that IL-17 could activate NF-κB signaling pathway, as well as MAPK signaling pathway^31^. Moreover, many other inflammatory mediators, like prostaglandin E2 (PGE2), COX2, nitric oxide (NO), could be upregulated by IL-17^19^,^32^. We demonstrated that IL-17 induced reactive astrocytes which were characterized by high-level expression of glial fibrillary acidic protein (GFAP). Reactive astrocytes are prominent in the cellular response to SCI and exhibit striking increases in GFAP immunoreactivity^33^,^34^.

Previously, it has been reported that VEGF significantly upregulate in SCI rats with allodynia^35^ and have a well-established role in axonal growth^36^. IL-17 in dedifferentiated OA chondrocytes causes the increasing expression of VEGF^13^. Here we showed that IL-17 stimulation induced the production and secretion of VEGF by reactive astrocytes. Subramanlam *et al*. had first reported IL-17 could trigger JAK/STAT pathways in human monocytic leukemia cell line^37^. In addition, previous study showed that STAT-3 promoted VEGF expression by a target gene, while STAT-1 negatively regulated VEGF expression in vascular smooth muscle cells^38^. To further address the role of IL-17 signaling in VEGF production, we confirmed that IL-17 increased STAT1, STAT3 and JAK2 expression and promoted their phosphorylation in a time-dependent manner. Knockdown of STAT1 or STAT3 effectively decreased VEGF expression at protein and mRNA levels. Furthermore, JAK2 inhibitor AG490 suppressed VEGF expression at protein and mRNA levels. And luciferase assays suggested that IL-17 modulated VEGF expression in a JAK/STAT-dependent manner. In addition, we also found that IL-17 treatment results in haemorrhage, loose structure and neutrophil infiltration in the spinal cord tissue, and residual urine volumes were remarkable increase at 14 day compared to SCI group. We also found IL-17 enhanced astrocyte reactivity, which GFAP immunoreactivity enhanced compared with SCI group, and damaged blood-spinal cord-barrier integrity *in vivo*. Taken together, these data indicated that the contribution of IL-17-induced activation of JAK/STAT signaling pathway to VEGF production. VEGF and its receptors can be expressed in astrocytes *in vitro and in vivo*, and hypoxia further elevate the level of VEGF in reactive astrocytes^39^,^40^. This newly found mechanism provides another understanding of spinal cord injury during inflammatory response and provides new insight into reactive astrocytes. This IL-17-JAK/STAT-VEGF axis could be a novel potential therapeutic target in spinal cord injury for the treatment of spinal cord injury.

## Methods

### Cell culture

The human astrocytoma cell line U251 was purchased from Shanghai Institute of Chinese Academy of Sciences (China) and maintained in RPMI-1640 (Gibco, USA), supplemented with 10% fetal bovine serum (FBS) at 37 °C in a humid incubator with 5% CO_2_.

### RNA extraction and quantitative real-time polymerase chain reaction (qPCR) analysis

Total RNA was extracted using the TRIzol^®^ reagent (Invitrogen, Carlsbad, USA). Reverse-transcribed complementary DNA was synthesized with the Prime-Script^®^ RT Reagent Kit (TaKaRa, Tokyo, Japan). qPCR analyses were performed with LightCycler 480 SYBR Green I Master (Roche, Welwyn Garden, Swiss).

### Animals

Adult, male, Sprague-Dawley (SD) rats weighing 180 to 220 g were provided by the Experimental Animal Center of Central South University and were housed at the animal facility of the Laboratory Animals Centre of Anhui Medical University. This study was carried out in strict accordance with the guidelines of the Care and Use of Laboratory Animals of the National Institutes of Health. All experimental protocols described in this study were approved by the Ethics Review Committee for Animal Experimentation of Anhui Medical University.

### Animal model of SCI

Anesthesia was induced with 4% isoflurane and maintained with 2% isoflurane in 98% O^2^. Throughout the procedure, the depth of sedation was monitored by an absent response to a toe pinch. A laminectomy was performed at the thoracic vertebra level 10 (T10) after shaving and cleaning in an incubator (37.5 °C) until fully recovered from the anesthesia and then each rat was housed individually. A moderate contusion injury was induced using a modified Allen’s weight drop apparatus (8 g weight at a vertical height of 40 mm, 8 g × 40 mm) on the spinal cord, as previously described^41^. Sham-operated animals were only subjected to laminectomy. After surgery, the muscles were sutured in layers and the skin incision was closed with 3-0 silk threads. Penicillin G (40,000 U, i. m.) was administrated daily for 3 days to prevent infection. Abdominal massage was conducted twice daily to help in the recovery of bladder function until full voluntary or autonomic voiding was obtained. Rats that died for any reasons were excluded from the experiment, and a new one was added to the study. The mortality rate of this study was approximately 2%.

### Experimental groups and interventions

The rats were randomly assigned to five groups (N = 6): sham-operated rats (sham group), IL-17-treated SCI rats (SCI + IL-17 group), IL-17 inhibitor-treated SCI rats (SCI + anti-IL-17 group), bevacizumabor-treated SCI rats (SCI + beva group), and SCI rats that received no treatment (SCI group). 1 ug/day of IL-17, 5 mg/kg/day bevacizumab and 25 mg/kg/day mouse anti-IL-17 antibody was administered by intraperitoneal injection 30 minutes after injury.

### Urine collection

Daily residual urine volumes were detected from morning volumes. To obtain urine from SCI rats at various times postinfection, animals were anesthetized with 2% inhaled isoflurane and administered 2 ml PBS intravenously via the tail vein to facilitate urine production. After 1 hour, urine was collected via transurethral catheterization.

### Behavioral assessment

Three rats from each group were subjected to locomotor activity evaluation at 1, 2, 3, 7 and 14 days post-injury using the Basso, Beattie, and Bresnahan (BBB) score method. Two independent and well-trained testers observed movement of each rat for 4 min and scored motor functions according to BBB scales^42^. The final score for each animal was obtained by averaging values from both investigators. Rats with perineal infections, limb wounds, or tail and foot grazing were eliminated from the test.

### HE staining

The 5 μm animal sections were made from the paraffin embedded blocks of the formalin-fixed spinal cord specimens, which were harvested from the animals from each group were subjected to routine HE staining and evaluated microscopically. Images were collected at 400X magnification.

### Tissue preparation

For the enzyme-linked immunosorbent assay (ELISA) and western blot, rats were sacrificed by transcardiac perfusion with cold PBS to eliminate RNA and protein expressed by blood cells. The spinal cord was immediately dissected on ice. Thereafter, 10-mm-long spinal cord segments containing the injury epicenter were removed as quickly as possible. The samples, except for the ELISA samples, were then flash-frozen and stored in liquid nitrogen for subsequent RNA and protein extraction. Spinal cord weight was subsequently measured using an electronic analytical balance. Three rats from each group were subjected to mouse IgG measurements from 72 h post-injury by ELISA.

### ELISA analysis

VEGF from U251 cells culture supernatants was quantified using Quantikine ELISA kits (R&D Systems) and performed according to the manufacturer’s instructions. The three spinal cords of each group at 72 h post-injury were used to detect cytokine protein levels by ELISA according to manufacturer’s instructions (Cusabio Biotech Co, Wuhan, China). All assays were performed in duplicates using recommended buffers, diluents, and substrates.

### Western blot

Proteins were separated on a 7.5% or 10% sodium dodecyl sulfate-polyacrylamide gel and transferred on to a nitrocellulose membrane (Bio-Rad, Hercules, USA). Then, the membrane was blocked with 5% non-fat milk and incubated with rabbit primary antibodies. The proteins were detected using enhanced chemiluminescence reagents (Thermo Scientific).

### Luciferase Reporter assays

The promoter of VEGF was amplified from the genomic DNA of HEK-293T and subcloned in pGL 3.0 luciferase reporter plasmid. The cells seeded into 96-well plates were transfected with pRL-CMV renilla luciferase reporter and the pGL 3.0 luciferase reporter plasmid. After 48 h, firefly and renilla luciferase activities were measured using a dual-luciferase reporter system.

### Adenovirus vector construction, infection and transfection

Oligonucleotides of shRNA were ordered from Sangon Biotech (Shanghai). HEK-293T cells were plated in culture dish. After HEK-293T cells attached to the plate overnight, the linearized DNA, the shuttle vector and pacAd vector were cotransfected into HEK-293T by Lipofectamine 2000 (Invitrogen). Viral lysates were harvested, purified and tittered. One day prior to infection, cells were seeded in plate. The next day, adenoviruses were added into the medium and infecting the cells.

### Immunohistochemistry

Albumin (Abcam), claudin-5 and gout anti-rat IgG antibodies (Santa Cruz Biotechnology) were used to evaluate BSCB integrity. CD68 and CD11b antibody were used to evaluate non-activated macrophages and microglia. GFAP antibody (Cell signaling) was used to evaluate astrocyte reactivity. Ki67 antibody (Abcam) wass used to evaluate cell proliferation. Sections were incubated in a hydrogen peroxide solution (0.3%) for 1 hour at room temperature. Bound antibodies were visualized using epifluorescence microscopy (Nikon Eclipse1000) or visualized using confocal microscopy (Zeiss 710 and LSM software).

### Statistical analysis

Results are presented as the means ± S.D. from at least 3 independent experiments. *P < 0.05 versus control, **P < 0.01 versus control, ^#^P < 0.01 versus PBS treatment. The statistical differences were calculated by the Student’s *t*-test or one-way ANOVA analysis of variance with Dunnett’s test.

## Additional Information

**How to cite this article:** You, T. *et al*. IL-17 induces reactive astrocytes and up-regulation of vascular endothelial growth factor (VEGF) through JAK/STAT signaling. *Sci. Rep.* **7**, 41779; doi: 10.1038/srep41779 (2017).

**Publisher's note:** Springer Nature remains neutral with regard to jurisdictional claims in published maps and institutional affiliations.

## Supplementary Material

Supplementary Information
