# Supplementary material for: IL-17 induces reactive astrocytes and up-regulation of vascular endothelial growth factor (VEGF) through JAK/STAT signaling
Source: Sci Rep. 2017 Mar 10;7:41779. doi: 10.1038/srep41779 (PMC5345044; doi:10.1038/srep41779)
Supplement: Supplementary Information [file srep41779-s1.pdf]

IL-17 Induces reactive astrocytes and up-regulation of vascular endothelial growth factor (VEGF) through JAK/STAT signaling

Tao You[1,2^], Yihui Bi[2^], Jun li[3], Mingkai Zhang[2], Xuezhou Chen[2], Keke Zhang[2], Jun Li [1*]

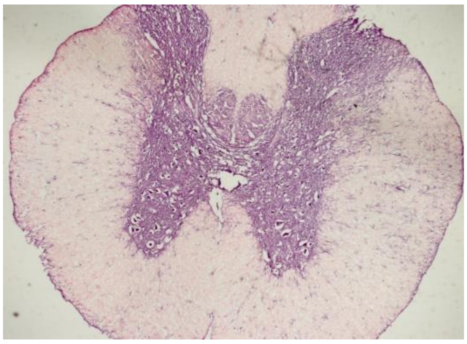

sham

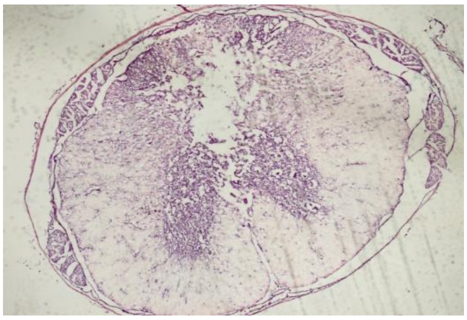

SCI

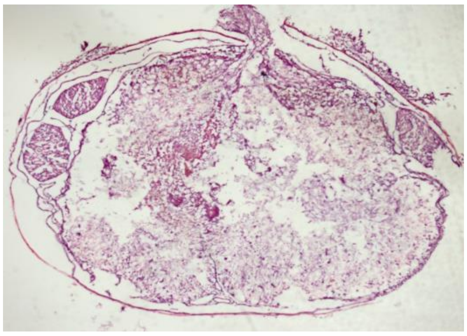

IL-17

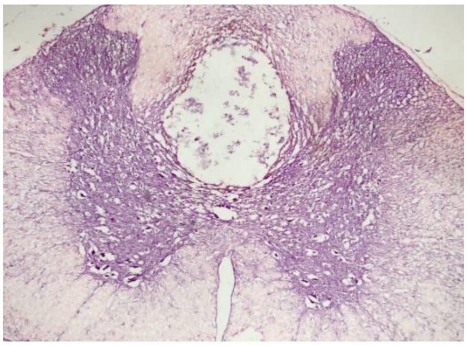

Beva

Figure legend. HE staining from various groups were used to visualize tissue changes.
